# Obesity accelerates aging: Mechanisms and therapeutic implications

**DOI:** 10.1016/j.gendis.2025.101980

**Published:** 2025-12-11

**Authors:** Rui Zhang, Linlin Liu, Xiaoman Shi, Yanming Ren

**Affiliations:** a Department of Neurosurgery, West China Hospital, Sichuan University, Chengdu, Sichuan 610041, China; b The First College of Clinical Medicine, Chongqing Medical University, Chongqing 400016, China

**Keywords:** Aging, Aging-related diseases, Anti-obesity therapies, Obesity, Underlying mechanisms

## Abstract

Global aging is increasing, and both aging and age-related diseases have emerged as significant public health challenges. According to previous literature, aging is characterized by multiple hallmarks, such as systemic inflammation, telomere depletion, mitochondrial dysfunction, and others. Obesity is a chronic and complex disease. Notably, obesity can accelerate the aging process and shares several similar features with aging. Therefore, this review systematically summarizes the relationship between obesity and aging, and discusses the great potential of anti-obesity therapies in combating aging and aging-related diseases.

## Introduction

Aging is a fundamental biological phenomenon observed across all species, encompassing a range of processes from molecular alterations to the aging of organelles and cells. As senescent cells accumulate, aging manifests progressively within tissues, organs, and the organism as a whole. This process is characterized by deteriorating health conditions, metabolic alterations, diminished functionality of tissues and organs, structural degradation, and reduced adaptability. To explore how to delay aging effectively, scientists have summarized twelve aging characteristics that may be slowed, stopped, or reversed through intervention: genomic instability, telomere depletion, epigenetic changes, loss of protein balance, loss of autophagy, deregulated nutrient sensing, mitochondrial dysfunction, cellular aging, stem cell depletion, changes in intercellular communication, chronic inflammation, and dysbiosis.^1^

The accumulation of these characteristics is associated with an increased prevalence of various age-related diseases. Research indicates that interventions aimed at slowing the aging process can postpone the onset and progression of various diseases in numerous rodent models. Consequently, such aging interventions may offer novel strategies for the comprehensive treatment of multimorbidity.^2^ Over the past few decades, several potential candidates for aging modulation have been identified. Notably, metformin was the first pharmacological agent to be evaluated for its effects on aging in a large-scale clinical trial.^3^^,^^4^ Originally utilized for the management of diabetes, metformin has subsequently been shown to effectively extend lifespan across various species, including worms, mice, and monkeys.^5^, ^6^, ^7^, ^8^

Furthermore, senolytic agents that target senescent cells have been shown to attenuate the senescence-associated secretory phenotype (SASP) by eliminating these aging cells, thereby contributing to an extension of healthy lifespan and a reduction in age-related pathologies.^9^, ^10^, ^11^, ^12^, ^13^, ^14^ Additionally, researchers have identified compounds such as nicotinamide nucleoside and nicotinamide mononucleotide as precursors to nicotinamide adenine dinucleotide (NAD^+^). These compounds are capable of restoring youthful NAD^+^ levels within cells, facilitating DNA repair processes in the nucleus, enhancing cellular energy metabolism, and exhibiting anti-aging effects.^15^^,^^16^ Despite the extensive research dedicated to decelerating the aging process and ameliorating age-related diseases, there remains a lack of compelling clinical trials that validate the efficacy of these interventions for human application. Consequently, there is significant potential for the advancement of novel pharmacological agents aimed at addressing the aging process. Notably, the new use of old drugs is undoubtedly the most time-saving and cost-effective way.

Obesity and aging are both conditions that lead to serious health problems and increase the risk of disease and death. Importantly, a quarter of Canadian adults are obese or overweight base on body mass index (BMI), and this condition becomes more common as they get older.^17^ There are intricate and multifaceted connections between obesity and aging. Obesity is associated with a variety of chronic and degenerative diseases, such as type 2 diabetes, osteoarthritis, cancer, and cardiovascular and renal dysfunction, and may lead to premature aging.^18^ A large amount of research evidence suggests that obesity can affect the accumulation of various aging biomarkers, including telomere shortening, epigenetic changes, disruptions in protein homeostasis, mitochondrial dysfunction, cellular senescence, stem cell depletion, and alterations in intercellular communication. Meanwhile, interventions aimed at extending health and lifespan, such as calorie restriction and exercise, are associated with reducing obesity.^19^ Therefore, this review provides a detailed summary of the impact of obesity on the aging process and discusses the great potential of anti-obesity therapies in combating aging and aging-related diseases.

## Obesity accelerates aging

**Figure 1:**
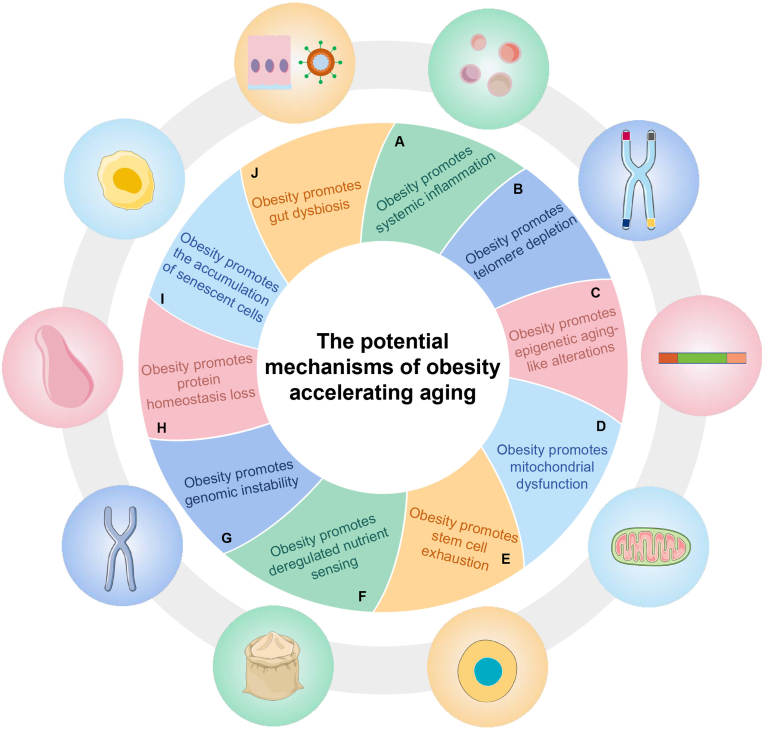
Ten key processes underlying obesity-accelerated aging, including system inflammation, telomere depletion, epigenetic aging-like alterations, mitochondrial dysfunction, stem cell exhaustion, deregulated nutrient sensing, genomic instability, homeostasis loss, the accumulation of senescent cells, and gut dysbiosis.

Obesity is characterized by an excessive accumulation of body fat that adversely affects health. According to the World Health Organization (WHO), overweight in adults is characterized by a BMI of 25 kg/m^2^ or higher, while obesity is defined as a BMI of 30 kg/m^2^ or above. Research has shown that obesity leads to higher mortality rates, with the lowest mortality rate occurring when the BMI is between 20.0 kg/m^2^ and 25.0 kg/m^2^. As the BMI increases within the overweight range, the mortality rate gradually increases significantly.^20^^,^^21^ The increased mortality rate may be related to the increased risk of many aging-related diseases caused by obesity.^18^^,^^22^^,^^23^ Moreover, obesity and aging share some similar markers, such as changes in metabolic regulation, insulin resistance (IR), inflammation, and impaired immune function. This suggests that there may be significant overlap between the mechanisms that promote these two processes. Aging can occur at different rates, depending on the accumulation of damage or the decline in resistance function,^24^ whereas obesity is recognized as a significant contributor to the acceleration of the aging process (Fig. 1).^25^

### Obesity promotes systemic inflammation

The persistent chronic inflammatory state in elderly individuals is one of the immune changes that occur during the aging process.^26^ The age-associated elevation of proinflammatory mediators, coupled with a reduction in anti-inflammatory factors, is considered a contributing factor to the onset of numerous age-related diseases.^27^, ^28^, ^29^ In obese people, inflammation occurs in various tissues, including adipose tissue, skeletal muscle, liver, intestine, pancreatic islets, and brain, with the most prominent being adipose tissue, as a result of the accumulation of immune cells and increased inflammation polarization.^30^ The alteration of immune cells in adipose tissue serves as a critical factor in promoting chronic tissue inflammation. Both innate and adaptive immune cells undergo significant changes in the context of obesity. Notably, macrophages represent the most abundant immune cell population in adipose tissue under conditions of both genetic and diet-induced obesity.^31^ Adipocytes secrete elevated levels of chemotactic molecules, such as monocyte chemoattractant protein-1 (MCP-1), which facilitate the recruitment of monocytes from peripheral blood into adipose tissue, where they subsequently differentiate into macrophages. Furthermore, MCP-1 stimulates the local proliferation of resident adipose tissue macrophages. Consequently, the proportion of macrophages in total immune cells in the adipose tissue of obese individuals increases from 10% to more than 40%.^32^ Simultaneously, during the progression of obesity, macrophages exhibit pronounced M1 polarization, leading to the release of proinflammatory cytokines. The increase in the number of macrophages and the increased ratio of M1 to M2 macrophages serve as markers associated with adipose tissue inflammation and are closely linked to the development of IR and metabolic disorders.^32^ Elevated chemokine ligand (CXCL-2) release from adipose tissue promoted neutrophil infiltration. Meanwhile, the high-fat diet (HFD) induced a 20-fold increase in the neutrophil content in the adipose tissue of obese mice.^33^ Neutrophils may promote adipose tissue inflammation by secreting elastase, peroxidase and interleukin-1β (IL-1β).^34^ Conversely, obesity decreases the number of adipose tissue eosinophils, leading to decreased insulin sensitivity.^35^, ^36^, ^37^ In addition, dendritic cells are also significantly increased in the adipose tissue of obese mice and humans and promote T-cell differentiation into Th17 cells, which have indeed been found to be significantly increased in number in overweight and obese patients.^38^ Mast cells are also elevated and activated in the adipose tissue of obese mice and humans and may promote adipose tissue inflammation by producing various inflammatory mediators, such as interleukin-6 (IL-6) and interferon gamma (IFN-γ).^34^ Innate lymphoid cells (ILCs) are recognized as modulators and respond to the inflammatory response to obesity.^39^ Mature ILCs can be further categorized into 3 subpopulations based on the differential expression of transcription factors, cell surface markers, and effector cytokines: group 1 ILC (ILC1), group 2 ILC (ILC2), and group 3 ILC (ILC3).^40^, ^41^, ^42^, ^43^ Obesity promotes ILC1 proliferation in adipose tissue, whereas ILC1-derived IFN-γ induces macrophage M1-like polarization and further enhances adipose tissue inflammation, promoting obesity-associated IR.^34^^,^^44^^,^^45^

Among adaptive immune cells, T cells constitute the largest proportion of adipose tissue, with CD4^+^ and CD8^+^ T cells being the most dominant. Obesity increases the proportion of CD8^+^ T cell populations.^46^ CD8^+^ T cells interact with local macrophages, triggering macrophage differentiation, activation, and migration, which triggers inflammation and leads to IR.^39^ CD8^+^ T-cell depletion inhibits inflammatory macrophage infiltration and downstream inflammatory cascade responses.^47^ In contrast, Treg cells in adipose tissue are reduced by up to 70% in HFD-induced obese mice.^48^ The reduction in Treg populations significantly increases the populations of macrophage and effector T cell within adipose tissue.^48^ This shift towards a pro-inflammatory cell population is most prevalent in the visceral adipose tissue and contributes to the inflammation associated with obesity.^39^

B lymphocytes, another type of adaptive immune cell, accumulate in the visceral adipose tissue of diet-induced obese mice and have the direct ability to promote pro-inflammatory T cell function and secrete a pro-inflammatory cytokine profile.^49^^,^^50^ Conversely, the absence of B cells in obese mice significantly reduces systemic inflammation and IR.^49^ In addition to adipose tissue, obesity effectively induces chronic inflammation. In the digestive system, the liver, which is primarily responsible for glucose production during fasting, is a key site of metabolic homeostasis and inflammation in obesity. Obesity leads to a significant increase in hepatic macrophage infiltration, as well as local production of inflammatory chemokines and cytokines.^51^ The nervous system is similarly disrupted by obesity. Studies have shown that microglial activation and hypothalamic inflammation are triggered three days after exposure to a HFD, even before any changes in body weight occur.^52^ Moreover, because obesity-induced persistent reprogramming of the innate immune system continues long after metabolic abnormalities are normalized, patients with a history of obesity are also susceptible to neuroinflammation due to reprogramming of mononuclear phagocytes.^53^ In the male reproductive system, obesity can drive testicular inflammation, leading to reproductive dysfunction.^54^ Additionally, chronic inflammation caused by obesity is a key driver of urinary system diseases, such as chronic kidney disease.^55^ Obesity also induces airway inflammation, increasing the risk and severity of respiratory diseases, including allergic asthma.^56^ Overall, obesity exacerbates immune cell metabolic dysregulation and promotes the accumulation of multiple pro-inflammatory immune cells while reducing the number of anti-inflammatory cells, which drives sustained low-grade inflammation in the body and promotes metabolic disease.

### Obesity promotes telomere depletion

Telomeres are DNA‒protein complexes at the ends of human eukaryotic cell chromosomes and are typically shorten with age.^57^ Replication of DNA polymerases cannot fully replicate the telomeric regions of eukaryotic DNA. Therefore, after several rounds of cell division, telomeres significantly shorten, leading to genomic instability and ultimately resulting in cell apoptosis or senescence which promotes overall aging and age-related diseases.^1^^,^^58^

Obesity accelerates telomere shortening. Multiple studies have shown a negative correlation between obesity, especially central obesity, and telomere length.^59^, ^60^, ^61^ Oxidative stress and inflammation are potential mechanisms by which obesity leads to telomere shortening.^60^ Moreover, regardless of age, weight gain and obesity both promote telomere loss.^62^ Importantly, the telomere length of young obese individuals is similar to that of elderly lean and elderly obese patients, with the shortest telomeres observed in elderly obese subjects.^63^ Weight loss intervention not only prevents telomere shortening but also plays an important role in telomere elongation. Telomere elongation is positively correlated with weight loss, and the elongation rate increases with increasing weight loss.^64^ In a study on the effect of calorie restriction-induced weight loss on telomere length in the rectal mucosa of obese men, greater weight and fat loss were associated with a greater increase in telomere length, and telomere length was directly negatively correlated with changes in adipose tissue.^65^

### Obesity promotes epigenetic aging-like alterations

Epigenetics refers to changes in phenotype or gene expression caused by mechanisms other than DNA sequence alterations.^66^ Epigenetic mechanisms include DNA methylation, histone modification, chromatin remodeling, and transcriptional alterations of non-coding RNA (ncRNA).^67^ The epigenome of senescent cells shows a loss of chromatin rigidity, increased entropy, epigenomic disorder, reduced compartmentalization, convergent changes in genome-wide epigenetic features, and decreased polarity.^68^ These changes can affect gene expression and other cellular biological processes, leading to the occurrence and development of various age-related diseases.^1^

Obesity promotes epigenetic aging-like alterations and contributes to an elevated risk of disease in old individuals.^69^ Studies have shown that BMI is positively correlated with epigenetic aging in middle-aged individuals.^70^ An increase in BMI is associated with changes in the methylation of specific genes, and DNA methylation changes may also lead to obesity.^70^, ^71^, ^72^, ^73^ Obesity induces chromatin modifications that lead to reduced methylation of the melanocortin-4 receptor (MC4R) gene, which regulates body weight.^74^ Similar chromatin changes are also observed during cellular senescence.^74^ In addition, changes in histone modifications correlate with altered expression of genes involved in fat accumulation and metabolic processes, while low histone acetylation is also observed in senescent cells.^74^ However, such epigenetic alterations caused by obesity also show tissue specificity. For instance, obesity accelerates epigenetic changes related to human liver aging, with a 2.7-point increase in BMI leading to a 10-year acceleration in epigenetic age but not in blood.^75^, ^76^, ^77^

### Obesity promotes mitochondrial dysfunction

Mitochondria are double-membrane organelles, with the inner membrane forming folds called cristae that extend into the mitochondrial matrix. Mitochondria not only serve as the powerhouses of the cell but also as constitute potential triggers for inflammation and cell death. With aging, mitochondria within cells gradually exhibit instability of respiratory chain complexes, decreased membrane potential, increased proton leakage, and accumulation of mitochondrial DNA (mtDNA) mutations, leading to a significant decline in mitochondrial function.^78^^,^^79^ This mitochondrial dysfunction further promotes aging.^1^ Obesity is also associated with mitochondrial dysfunction. In both rodent and human adipocytes, obesity induces decreased expression of mtDNA, its transcripts, and oxidative phosphorylation (OXPHOS) subunits, resulting in reduced mitochondrial biosynthesis.^80^^,^^81^ Obesity diminishes mitochondrial oxidative capacity by downregulating multiple metabolic pathways, such as fatty acid β-oxidation (FAO), the tricarboxylic acid cycle (TCA), ketogenesis, ketolysis, and branched-chain amino acid (BCAA) degradation pathways.^80^^,^^81^ Additionally, obesity causes increased mitochondrial fission, decreased mitochondrial fusion, reduced mitochondrial respiratory capacity, and decreased adenosine triphosphate (ATP) content in the skeletal muscle of mice and humans, indicating impaired mitochondrial dynamics.^82^, ^83^, ^84^ Related studies have shown that the specific mechanism may involve the increase of fission-related protein fission 1 homolog protein (Fis1) and dynamin-related protein 1 (Drp1), and the decrease of mitochondrial fusion protein 2 (Mfn2) and electron transport chain efficiency.^82^, ^83^, ^84^ In addition to the mitochondrial abnormalities observed in adipocytes and skeletal muscle cells, mitochondrial dysfunction has also been detected in other tissues of obese individuals. In the heart, obesity promotes mitochondrial changes related to aging, including changes in mitochondrial morphology, loss of mitochondrial contents, reduced mitochondrial respiration, and disrupted mitochondrial dynamics, leading to impaired cardiac energy metabolism.^85^ In the liver, diet-induced obesity also leads to an increased mitochondrial fission rate, as well as decreased expression of optic atrophy 1 (OPA1) and Mfn2, causing mitochondrial dysfunction.^86^^,^^87^

### Obesity promotes stem cell exhaustion

Stem cells are progenitor cells with the potential for self-renewal and multi-directional differentiation. Both the regenerative capacity of stem cells and their proportion among total cells decline with aging. It has been reported that the proportion of bone marrow mesenchymal stem cells during aging decreases by nearly 200 times compared to that at birth.^88^ Moreover, a series of changes occur in the tissues and cells of the organism during aging, including the accumulation of DNA damage, chronic inflammation, mitochondrial dysfunction, alterations in autophagy, and epigenetic changes, all of which can lead to stem cell aging and exhaustion.^89^ The decline of stem cells is an important cellular driver of the pathophysiology associated with aging in various tissues.

Obesity, similar to the aging process, significantly interferes with the function and quantity of stem cells. The proportion of adipose-derived stem cells (ASCs) and precursor cells in the subcutaneous adipose tissue of obese patients is significantly reduced.^90^^,^^91^ ASCs isolated from obese patients show a significant decrease in proliferation ability, reduced differentiation potential and angiogenic capacity, as well as a decline in self-renewal ability.^91^^,^^92^ Within ASCs, telomerase activity and telomere length are altered,^93^ mitochondrial content abnormally increases, and function is impaired, leading to an increase in reactive oxygen species (ROS). At the gene expression level, ASCs from obese individuals show a decrease in the expression of stem cell-related genes, an increase in the expression of adipocyte differentiation-related genes, and an upregulation of inflammatory genes.^94^ These findings indicate a decline in the stemness function of ASCs in obese patients and an increase in cellular oxidative stress. In mice, obesity has also been found to promote a decrease in the expression of stemness-related genes in ASCs, a significant increase in senescence-related phenotypes, changes in cell cycle distribution, and a reduction in the number of cells expressing pluripotency markers.^95^ Meanwhile, it has been discovered in research that merely culturing ASCs with plasma sourced from obese patients results in decreased cell proliferation capacity, G2/M cycle arrest, upregulation of cyclin-dependent kinase inhibitor 1a (p21) and cyclin-dependent kinase inhibitor 2A (p16), increased activity of senescence-associated β-galactosidase (SA-β-gal), and increased levels of IL-6 and interleukin-8 (IL-8).^96^ In addition to ASCs, obesity affects the quantity and function of various stem cells throughout the body. Circulating endothelial progenitor cells in obese patients are reduced in number and have a weakened colony-forming ability, and the impairment of endothelial progenitor cell quantity and function may lead to obesity-related cardiovascular risks.^97^ In mice, HFD-induced obesity promotes enhanced differentiation of bone marrow progenitor cells into adipocytes, thereby causing progenitor cell exhaustion and a decrease in osteoblast formation and bone formation.^98^ Mice fed with HFD presented a reduction in the number of neural stem cells and a decrease in their differentiation ability in the hypothalamus.^99^

### Obesity promotes deregulated nutrient sensing

Nutrient sensing refers to the ability of cells to recognize and respond to energy substrates such as glucose, fatty acids, and ketone bodies. Three key nutrient-sensing pathways are the insulin/insulin-like growth factor-1 (IGF-1) signaling pathway, the mammalian target of rapamycin (mTOR) pathway, and the adenosine 5′-monophosphate (AMP)-activated protein kinase (AMPK) pathway. In mammals, insulin/IGF-1 signaling is an important coordinator of nutrient availability with energy homeostasis and metabolic processes, which can initiate signal transduction through the phosphatidylinositol 3-kinase/protein kinase B (PI3K/Akt) pathway and then phosphorylate many targets to regulate the activity of the mTOR complex (mTORC).^100^ The mTORC1 network is a central regulator of cellular activities, responding to nutrients (including glucose and amino acids) as well as stressors such as hypoxia and low energy to regulate the activity of many proteins.^1^ The mammalian AMPK pathway is triggered by a decline in the cellular energy status and activated by an increase in the adenosine monophosphate (AMP)/ATP and adenosine diphosphate (ADP)/ATP ratios.^101^ Obesity exacerbates age-related energy metabolism disorders. The insulin/IGF-1 signaling pathway and the mTOR pathway are regarded as “accelerators” of the aging process.^102^^,^^103^ Increasing evidence indicates that these pathways are also over-activated in obesity. IGF1 expression is increased in the adipose tissue of obese patients and animal obesity models.^104^ Elevated IGF1 promotes adipogenesis gene expression and reduces energy expenditure by activating Akt, which is the mammalian target of the mTOR pathway in adipose tissue, leading to obesity in mice.^105^ In a genetic mouse model with high IGF1 secretion, downregulation of IGF1 normalized the expression of adipogenic genes, restored abnormal energy metabolism, and alleviated the obese phenotype.^104^ In the tissues of obese and HFD-fed rodents, mTORC1 is chronically over-activated.^106^^,^^107^ Moreover, S6K1, a direct downstream target of mTOR, is also over-activated in the adipose tissue, liver, and muscle of different genetic mouse models of obesity.^108^ The expression level and activity of ribosomal protein S6 kinase 1 (S6K1) in the visceral fat of obese patients are also significantly increased.^109^ Interventions on the mTOR pathway significantly affect the development of obesity. Studies have shown that S6K1 knockout mice are resistant to age- and diet-induced obesity, showing increased lipolysis and reduced adipose tissue mass.^106^^,^^110^ Additionally, inhibiting the mTOR pathway can also improve various negative effects brought by aging and obesity.^85^^,^^111^

Activation of the AMPK pathway is regarded as one of the effective ways to delay aging, but AMPK is significantly inhibited by obesity. Under physiological conditions, AMPK inhibits lipid synthetic metabolism and activates lipid catabolic metabolism. Clinical studies have shown that the activation of AMPK in the skeletal muscle of obese and diabetic patients is impaired.^112^ In mice, HFD-induced obesity leads to a significant reduction in AMPK activity in white adipose tissue, paraventricular nucleus, heart and liver.^113^, ^114^, ^115^ Conversely, in mice, intervention to activate the AMPK pathway can promote the browning of white adipose tissue, inhibit adipogenesis and inflammation, and improve obesity in HFD-fed mice.^116^^,^^117^

### Obesity promotes genomic instability

The integrity and stability of the genome are generally threatened by exogenous chemicals, physical and biological agents, as well as endogenous challenges. The extensive genetic damage caused by these exogenous or endogenous damage sources includes point mutations, deletions, translocations, telomere shortening, single-strand and double-strand breaks, chromosomal rearrangements, nuclear structural defects, and gene disruption caused by viral or transposon integration.^118^ All these molecular alterations and the resulting genomic mosaicism can lead to both normal and pathological aging.^119^ Obese individuals are prone to various types of DNA damage, such as double-strand breaks, single-strand breaks, and oxidized bases. As mentioned earlier, oxidative stress and inflammation, which can induce DNA damage and inhibit DNA repair mechanisms, are common in obese individuals.^120^ Studies have shown that obesity activates macrophages and induces them to secrete multiple cytokines, such as tumor necrosis factor-α (TNF-α) and IL-6, which can induce DNA damage in non-target tissues far from the site of inflammation.^121^^,^^122^ Clinical studies have revealed that DNA damage in the lymphocytes of obese patients is approximately twice as high as that in the lymphocytes of normal-weight subjects, and there is a direct correlation between BMI and DNA damage.^123^^,^^124^ In adolescents and children, DNA damage caused by obesity is more significant. As long as individuals are overweight, regardless of their BMI, lymphocyte DNA damage will occur.^124^^,^^125^ Obesity not only directly causes various types of DNA damage but also inhibits the functioning of DNA repair pathways. In young women, BMI is negatively correlated with the ability of nucleotide excision repair (NER).^126^ This may be related to the expression changes of certain genes involved in the repair process and the functional blockage of DNA repair enzymes caused by obesity.^127^, ^128^, ^129^ Interventions for obesity also reduce the degree of DNA damage. After weight loss induced by a low-calorie diet, a significant decrease in DNA damage levels was observed.^65^^,^^130^ After bariatric surgery in morbidly obese patients, an improvement in genomic stability was also observed.^131^

### Obesity promotes loss of protein homeostasis loss

The loss of protein homeostasis in senescent cells can be caused by multiple factors. The three main factors are as follows: first, an increase in the production of misfolded or incompletely translated proteins; second, the deceleration of the translation elongation rate and the accumulation of protein oxidative damage increasingly distract the attention of molecular chaperones, preventing them from folding the healthy proteins necessary for cells; and lastly, the failure of mechanisms ensuring protein quality control, such as the reduced function of the unfolded protein response (UPR) in the endoplasmic reticulum (ER).^132^^,^^133^ When the stability of correctly folded proteins is compromised or the ability of the proteasome or lysosome to degrade misfolded proteins becomes insufficient, the protein homeostasis network collapses, thereby accelerating aging.^134^ Obesity can induce long-term or chronic UPR.^135^ The ER controls the correct folding of polypeptides and proteins through various chaperones and enzymes. When misfolded/unfolded proteins accumulate in the ER lumen, ER stress is induced. A series of signal cascades induced by ER stress, known as the UPR, are triggered.^17^ Interventions that reduce ER stress can extend the healthy lifespan of mammals.^136^ Significantly increased ER stress markers have been found in genetically obese mice and HFD-fed obese mice,^137^ especially in the liver and adipose tissue.^138^ In addition to exerting adverse effects on the UPR pathway induced by ER stress, numerous studies have shown that obesity also impacts the protein degradation pathway. The two major quality control systems responsible for protein and organelle degradation in eukaryotic cells are the ubiquitin‒proteasome system (UPS) and autophagy.^139^ The influence of obesity on the protein degradation pathway is more complex. The levels of ubiquitin in the plasma of obese patients and 20S proteasomes in red blood cells are significantly reduced, and they are negatively correlated with an individual's BMI.^140^ In genetic and dietary model mice with obesity and diabetes, total proteasome activity in the gastrocnemius muscle, anterior tibial muscle, and liver is significantly decreased.^141^^,^^142^ However, some studies have reported that proteasome activity is enhanced in the muscles of the obese diabetic db/db mouse model.^143^ Human skeletal muscle cells from obese patients also show elevated proteasome activity. Therefore, the proteasome activity caused by obesity still needs to be further explored in combination with tissue differences and various physiological conditions.

Obesity is associated with overnutrition, and both IR and hyperinsulinemia that it may cause are believed to contribute to the inhibition of autophagy.^144^ Preliminary studies on obese mice have shown that autophagic activity in the liver is significantly downregulated, which is related to the decreased expression of autophagy protein 5 (ATG5) and autophagy protein 7 (ATG7) and the subsequent inhibition of autophagosome biogenesis, as well as defects in the fusion of autophagosomes and lysosomes.^145^^,^^146^ However, other studies have shown that autophagy levels in the adipose tissue of obese patients, especially omental adipose tissue, are upregulated, with increased microtubule-associated protein 1 light chain 3 (LC3) levels in adipocytes and an increased number of autophagosomes, and are associated with higher BMI, greater omental visceral fat distribution, larger adipocyte diameter, and higher expression levels of autophagy gene ATG5 mRNA.^147^ Human skeletal muscle cells from obese patients also show increased autophagic flux.^148^ ER stress, inflammation, and oxidative stress caused by obesity can all induce autophagy through multiple mechanisms.^149^^,^^150^ In this case, autophagy induction can be regarded as a defense mechanism to coordinate and maintain cellular homeostasis under obesity-related stress. Therefore, the changes in autophagy levels throughout the development of obesity may be a dynamic process with tissue specificity.

### Obesity promotes the accumulation of senescent cells

Cellular senescence is a response to acute or chronic damage. Focal or tissue-specific accumulation of senescent cells occurs in many diseases. Continuous genetic or pharmacological elimination of senescent cells can extend the healthy lifespan and lifespan of naturally aging mice, effectively demonstrating the causal role of cellular senescence in aging.^151^

The number of senescent cells in adipose tissue increases with aging and obesity.^152^, ^153^, ^154^ The proportion of senescence-associated β-galactosidase-positive cells (SA β-gal^+^ cells) in preadipocytes and endothelial cells isolated from obese individuals is higher; and BMI is positively correlated with SA β-gal ^+^ activity and p53 in adipose tissue.^152^^,^^155^ In a diet-induced obesity mouse model, senescent CD4^+^ T cells and macrophages also accumulate in visceral adipose tissue.^156^ Cellular senescence caused by obesity is not limited to adipose tissue but also occurs in distant organs such as the brain. In HFD-fed mice, glial cells near the lateral ventricle, an area where adult neurogenesis occurs, show lipid deposition and accumulation of senescent cells.^157^ After 12 months of HFD consumption in mice, pancreatic β-cell proliferation decreases, the number of senescent cells increases, and pro-inflammatory molecule secretion rises.^158^ The long-term accumulation of senescent β-cells induces metabolic dysfunction through the loss of β-cell mass and function, which impairs glucose tolerance and leads to IR.

### Obesity promotes gut dysbiosis

The gut microbiome is widely recognized as playing a vital role in human health and disease.^159^ These microbial communities not only participate in local gastrointestinal physiology but also contribute to fundamental immune regulation and metabolic homeostasis at the systemic level.^159^ Current research identifies the gut microbiome as an aging marker, with specific changes, including reduced diversity and decreased populations of beneficial bacteria such as *Lactobacillus*.^159^, ^160^, ^161^, ^162^ These changes may impair intestinal barrier integrity and compromise immune regulation. Microbes influence aging-related processes through their metabolites, including metabolism, immunity, energy homeostasis, and cellular repair.^159^, ^160^, ^161^, ^162^ Dysbiosis of the gut microbiota can exacerbate the aging process.

**Figure 2:**
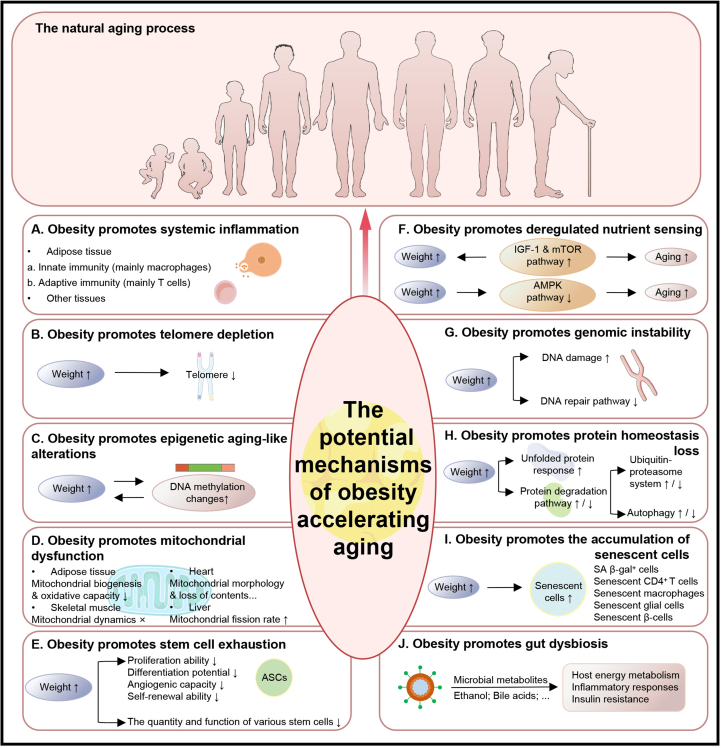
Underlying specific mechanisms linking obesity to accelerated aging. ASCs, adipose-derived stem cells; IGF-1, insulin/insulin-like growth factor-1; mTOR, mammalian target of rapamycin; AMPK, adenosine 5′-monophosphate-activated protein kinase; SA β-gal^+^ cells, senescence-associated β-galactosidase-positive cells.

Throughout the lifespan, obesity induces gut microbiota dysbiosis that deviates from normal aging patterns, characterized by reduced diversity and dominance of the *Bacteroidetes* or *Proteobacteria phyla*.^159^^,^^163^ Similar to how gut dysbiosis accelerates aging, obesity-associated microbiota alter host energy metabolism, inflammatory responses, and insulin resistance.^159^^,^^163^ Microbial metabolites, such as endogenous ethanol and bile acids, act as key signaling factors in this process.^159^ The increased production of compounds like endogenous ethanol by obesity-related microbiota disrupts mitochondrial function, promotes oxidative stress, and contributes to accelerated aging phenotypes.^159^ Concurrently, elevated lipopolysaccharide (LPS) levels enter the systemic circulation due to compromised intestinal barrier function. This low-grade endotoxemia induces chronic inflammation. Additionally, BMI is positively correlated with total plasma bile acid levels.^159^ Compared to lean individuals, obese individuals undergo a shift toward 12α-hydroxylation of bile acid synthesis pathways, altering their composition and ratios. Studies have demonstrated that lithocholic acid (LCA) administration improves aging-related phenotypes in mice^159^ by activating AMPK, which enhances mitochondrial content and respiratory capacity in muscle tissue while improving insulin resistance and increasing serum glucagon-like peptide-1 (GLP-1) levels.^159^ Thus, biliary acid imbalance in obese individuals may contribute to accelerated aging (Fig. 2).

## The association between anti-obesity therapies and anti-aging

Obesity undoubtedly accelerates aging and age-related diseases, and interventions for obesity also have a direct impact on the aging process. Studies have shown that transplanting senescent preadipocytes into young mice leads to physical dysfunction and shortens both healthspan and lifespan.^164^ Conversely, effective interventions that reduce adipose tissue mass, such as calorie restriction or surgical removal of visceral fat, can increase healthspan and lifespan.^165^, ^166^, ^167^, ^168^, ^169^ Therefore, considering the similarities between multiple phenotypes of obesity and aging, and the effectiveness of obesity interventions in delaying aging, anti-obesity therapy may hold great potential in delaying aging and combating age-related diseases.^170^

**Figure 3:**
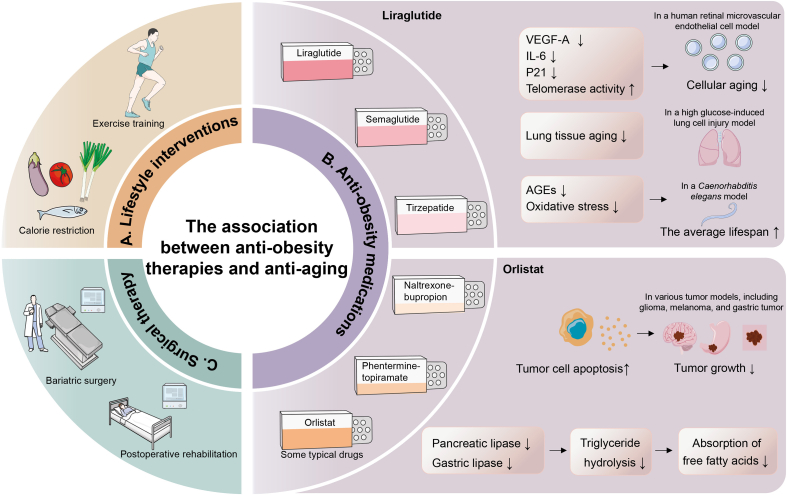
Current treatment options for obese patients primarily include lifestyle interventions, anti-obesity medications, and surgical therapy. Anti-obesity drugs have demonstrated significant potential in combating aging and aging-related diseases. VEGF-A, vascular endothelial growth factor-A; IL-6, interleukin-6; P21, cyclin-dependent kinase inhibitor 1a; AGEs, advanced glycation end products.

For obese patients, current treatment options primarily include lifestyle interventions, anti-obesity medications (Liraglutide, Semaglutide, Tirzepatide, Naltrexone-bupropion, Phentermine-topiramate, Orlistat, and so on), and surgical therapy.^171^^,^^172^ Among these, anti-obesity drugs have demonstrated significant potential in combating aging. A substantial body of research has explored their role in combating aging and treating age-related diseases. For instance, liraglutide, a classical glucagon-like peptide 1 receptor (GLP-1R) agonist, has shown anti-aging effects at the cellular, tissue, and individual levels, despite the lack of direct studies on its comprehensive impact on aging models or naturally aging individuals. In a human retinal microvascular endothelial cell model induced by high glucose, liraglutide inhibited the expression of vascular endothelial growth factor-A (VEGF-A), IL-6, and the aging marker protein p21, increased telomerase activity, and thereby delayed cellular aging.^173^ In a high glucose-induced lung cell injury model, liraglutide treatment significantly inhibited lung tissue aging.^174^ Additionally, in a *Caenorhabditis elegans* model cultured under conditions simulating clinical hyperglycemia, liraglutide significantly inhibited advanced glycation end products (AGEs) and oxidative stress, improved neuronal function, and ultimately increased the average lifespan of the worms by 9%.^175^ These findings suggest the potential of liraglutide in improving healthy lifespan and overall lifespan. Anti-obesity medications have also demonstrated potential in treating aging-related diseases. Orlistat is another example of such a drug. It inhibits pancreatic lipase and gastric lipase, reducing triglyceride hydrolysis and thereby suppressing the absorption of free fatty acids.^176^ In various tumor cells, including glioma cells,^177^ melanoma cells,^178^^,^^179^ and gastric tumor cells,^180^ orlistat inhibits tumor cell growth by promoting tumor cell apoptosis (Fig. 3).

However, this field still holds vast potential for further exploration. Overall, in light of prior research, we speculate that anti-obesity therapies all have the potential to be applied in the treatment of aging-related diseases and the coexistence of multiple diseases in elderly patients, thereby further achieving the extension of healthy lifespan and even lifespan. We are confident that this represents a promising avenue for future research.

## Conclusion

A large number of studies have found that obese patients exhibit various characteristics similar to aging, including systemic inflammation, telomere attrition, epigenetic alterations, mitochondrial dysfunction, stem cell exhaustion, dysregulation of nutrient sensing, genomic instability, protein homeostasis imbalance, cellular senescence, and gut dysbiosis. Obesity also increases the risk of age-related diseases and comorbidities.^181^ Undoubtedly, obesity is an accelerator of aging and aging-related diseases, andits intervention directly impacts the development of aging.^165^, ^166^, ^167^^,^^169^ However, the overlapping characteristics mentioned above merely indicate potential mechanisms by which obesity promotes aging, with the specific molecular mechanisms involved remaining unclear. These findings suggest that future efforts should focus on further exploring these mechanisms and validating them through targeted biological markers to advance precision medicine development. Such progress is expected to significantly contribute to addressing the challenges posed by an aging society.

## CRediT authorship contribution statement

**Rui Zhang:** Writing – original draft, Validation, Conceptualization. **Linlin Liu:** Writing – original draft, Visualization, Conceptualization. **Xiaoman Shi:** Writing – original draft, Validation, Conceptualization. **Yanming Ren:** Writing – review & editing, Supervision, Funding acquisition, Conceptualization.

## Funding

This work is supported by the 10.13039/501100001809National Natural Science Foundation of China (No. 82302627, 82472683).

## Conflict of interests

The authors declare that they have no known competing financial interests or personal relationships that could have appeared to influence the work reported in this paper.
